# Effects of Acupuncture-Point Stimulation on Perioperative Sleep Disorders: A Systematic Review with Meta-Analysis and Trial Sequential Analysis

**DOI:** 10.1155/2024/6763996

**Published:** 2024-01-04

**Authors:** Ying Liu, Yi Li, Meinv Liu, Meng Zhang, Jing Wang, Jianli Li

**Affiliations:** ^1^Department of Anaesthesiology, Hebei General Hospital, Shijiazhuang, China; ^2^Hebei Medical University, Shijiazhuang, China

## Abstract

**Background:**

Perioperative sleep disorders exert a severe adverse impact on postoperative recovery. Recently, some observational studies reported that acupuncture-point stimulation (APS) provided benefits for promoting perioperative sleep quality. However, the effects of APS on perioperative sleep disorders following general anesthesia have not been thoroughly assessed by any systematic study and meta-analysis. Therefore, we conducted this systematic review and meta-analysis to reveal the effects of APS on perioperative sleep disorders.

**Methods:**

Eight databases (Chinese: CNKI, VIP, CBM, and Wanfang; English: PubMed, Embase, Web of Science, and Cochrane Library) were thoroughly searched to find randomized controlled trials (RCTs) that indicated a link between APS and the occurrence of perioperative sleep disorders. We applied RevMan 5.4 (Cochrane Collaboration) and Stata 16.0 (Stata Corp) to conduct our meta-analysis. In addition, the trial sequential analysis (TSA) tool was utilized to estimate the validity and reliability of the data.

**Results:**

In this study, nine RCTs with 719 patients were conducted. Compared to the control group, APS significantly improved perioperative subjective sleep quality (SMD: −1.36; 95% CI: −1.71 to −1.01; *P* < 0.00001). Besides, it increased perioperative TST (preoperative period MD = 24.29, 95% CI: 6.4 to 42.18, *P* = 0.0008; postoperative period MD = 45.86, 95% CI: 30.00 to 61.71, *P* < 0.00001) and SE (preoperative MD = 3.62, 95% CI: 2.84 to 4.39, *P* < 0.00001; postoperative MD = 6.43, 95% CI: 0.95 to 11.73, *P* < 0.00001). The consequence of trial sequential analysis further confirmed the reliability of our meta-analysis results.

**Conclusion:**

According to the currently available evidence, APS could effectively improve perioperative sleep quality and play an essential role in decreasing the incidence of perioperative sleep disorders.

## 1. Introduction

Sleep is vital for overall health as it influences the quality of life and physical functioning [1]. Good sleep quality is a sign of wellbeing, whereas poor sleep quality raises comorbidity, mortality, and medical expenses [2]. Despite advances in operative and anesthetic procedures, sleep disorders continue to be a concern in the perioperative period. Perioperative sleep problems, as compared to household sleep disorders, have gained more attention recently, such as reduced nighttime sleep, fragmentation of sleep, and circadian rhythm disruptions at night after admission [3,C^4^]. The proportion of sleep disorders in hospitalized patients (64%–73.1%) is much higher than that in people at home (30.6%–41.2%) due to the surgical stress response, ward environment, and patients' underlying diseases [5–7]. Perioperative sleep problems were noted in several observational studies to be important risk factors for poor recovery [8] and were associated with anxiety, altered pain perception, and postoperative cognitive dysfunction (such as delirium), further deteriorating the patient's physical state [1, 6, 7, 9]. Moreover, it was suggested that perioperative sleep disorders might lead to postoperative fatigue, episodic hypoxemia, cardiovascular disorders, metabolic impairment, and immune disorders [10, 11]. Enhancing perioperative sleep quality is probably related to improved health, surgery effect, and patient safety [9]. Unfortunately, there are no specific therapeutic strategies for perioperative sleep disorders. Even though it was reported that pharmaceutical therapies, such as benzodiazepines and other sedating agents, might improve perioperative sleep quality, the potentially addictive nature and risk of tolerance restricted their clinical application [9].

In complementary and alternative medicine, acupuncture-point stimulation (APS) was employed to improve perioperative sleep quality [12]. According to the theory of traditional Chinese medicine (TCM), APS refers to stimulating the body's acupoints with various methods, including acupressure (manual stimulation), acupuncture (sterile needle stimulation), laser acupuncture, electrical stimulation (transcutaneous electrical acupoint stimulation and electrical acupuncture), magnetic acupuncture (stimulation with special equipment), moxibustion therapy, and auricular point pressing, with the goal of achieving therapeutic effects such as sleep improvement and pain management [13–18]. APS has been an integral part of traditional Chinese medicine treatment. Based on the traditional Chinese medicine meridian theory, the body's qi circulation is restored when target acupoints along meridians are stimulated [14]. It was reported that APS might lower the risk of poor sleep quality due to its considerable analgesic and sedative effects [19, 20]. Recently, a significant number of RCTs were carried out on the effects of APS on perioperative sleep disorders [8, 15]. Besides, a previous review concluded that EA might work to alter neurotransmitter concentrations and reduce the levels of norepinephrine and dopamine so as to enhance the quality of postoperative sleep quality after general anesthesia [12]. However, this study not only did not conduct a statistical assessment of the efficiency of EA on inpatients' sleep quality during the postoperative period but also did not examine preoperative sleep quality thoroughly [12].

Consequently, we carried out a quantitative meta-analysis to evaluate the effects of APS on perioperative sleep disorders, including the preoperative period and the postoperative period, to provide research-based evidence for clinical practice.

## 2. Materials and Methods

The PRISMA statement was implemented in the conception and presentation of our systematic review and meta-analysis, which was carried out to estimate the efficiency of APS in improving perioperative sleep quality [21]. The meta-analysis was registered in the PROSPERO database (registration number: CRD42023387848).

### 2.1. Search Strategy

We comprehensively searched the CNKI, Wanfang, VIP, CBM, Embase, PubMed, Cochrane Library, and Web of Science databases in both Chinese and English from the inception until December 2022. A basic search strategy was conducted using the following terms: “sleep,” “sleep^*∗*^,” “sleep quality,” “sleep disorder,” “sleep deprivation,” “acupuncture point,” “acupuncture points,” “acupoint^*∗*^,” “TEAS,” “EA,” “perioperative period,” “preoperative period^*∗*^,” “postoperative period^*∗*^,” and “surgery,”. The detailed search strategy is shown in Supplementary 1.

### 2.2. Inclusion and Exclusion Criteria

Nine qualified studies were published in Chinese and English between 2017 and 2022. Studies that matched the following criteria (PICOS) were eligible for our meta-analysis:

- Population: Patients receiving all kinds of surgery.
- Intervention: APS (there were no time constraints on interventions).
- Comparison: Usual care/nonacupuncture point stimulation.
- Outcomes: The Pittsburgh Sleep Quality Index (PSQI), Athens Insomnia Scale (AIS), and Insomnia Severity Index (ISI) were employed to assess the perioperative sleep quality as the primary outcome. In addition, total sleep time (TST) and sleep efficiency (SE) served as the secondary outcomes.
- Study Design: Randomized controlled trial.

The publications were excluded due to the following reasons: (1) inability to extract precise clinical data or access the complete text and (2) inclusion of reviews, animal experiments, case introductions, congress reports, etc.

### 2.3. Study Selection

EndNote X9 was utilized to remove duplicate articles and organize the references. Two writers independently assessed the abstracts and titles for initial evaluation after removing duplicates. If one study was determined to be qualified, we would download the whole text and carry out detailed screening. When the two authors came to an understanding, data extraction and analysis were performed on the qualified research. A third reviewer solved disagreements during selection.

### 2.4. Data Extraction

Essential characteristics of the included RCTs were independently extracted by different researchers using a designed data extraction framework. In the special data extraction table, primary writer, publication date, sample size, study design, surgery type, intervention, and reported outcome type (sleep quality assessment methods) were collected. If any material in the report needed to be clarified, an attempt was made to consult the original study's author. The collected data were considered for a systematic review and meta-analysis to assess the effects of APS on perioperative sleep disorders.

### 2.5. Assessment of the Risk of Bias

The Cochrane risk of bias assessment was used to evaluate the RCTs' methodological quality in seven distinct aspects, including (I) random sequence generation, (II) allocation concealment, (III) blinding of participants and personnel, (IV) blinding of outcome assessment, (V) incomplete outcome data, (VI) selective reporting, and (VII) other bias [22]. In this method, risk levels were classified as “high risk,” “uncertain risk,” and “low risk.” Significantly, the APS operator could not be blindfolded due to the unique nature of APS therapy and only participants and result measurement personnel might be. Two researchers separately conducted the assessment; in the event of a dispute, a third evaluator was asked to mediate.

### 2.6. Data Synthesis and Analysis

Our meta-analysis was synthesized to quantitatively summarize the qualified studies using ReviewManager software version 5.4. The efficiency of APS on perioperative sleep disorders was estimated by the following continuous outcomes reported as mean differences (MDs) with 95% CIs: PSQI, AIS, or ISI, and TST and SE. Generally, MD with 95% confidence intervals (CIs) was deemed to be suitable for continuous outcomes. However, the standardized mean difference (SMD) should be computed when the same continuous result was examined using multiple assessment tools. Results were considered significant for *P* values under 0.05. The chi^2^ test and *I*^2^ statistic were employed to assess study heterogeneity. If statistical homogeneity (*P* > 0.10; *I*^2^ < 50%) was observed in each trial, the fixed-effect model was employed for data analysis. If there was statistical heterogeneity between trials (*P* < 0.10; *I*^2^ > 50%), the source of heterogeneity was examined using a random-effects model in conjunction with a subgroup or sensitivity analysis. Sensitivity analyses were carried out for the included studies to determine which RCT impacted the overall results. Descriptive analysis was used if heterogeneity was too considerable for a meta-analysis. In addition, we performed the trial sequential analysis with TSA program version 0.9.5.10 beta to ascertain the statistical reliability of the data.

## 3. Results

### 3.1. Literature Search

**Figure 1:**
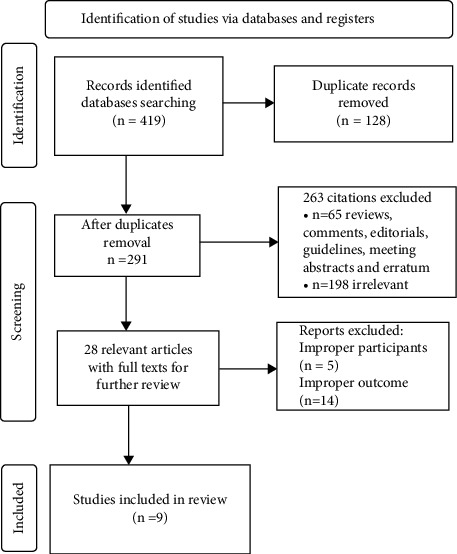
Flowchart for study selection based on the PRISMA study flow diagram.

A total of 419 studies were obtained from 8 databases: CNKI, Wanfang, VIP, CBM, Embase, PubMed, Cochrane Library, and Web of Science. 128 duplicate studies were identified and excluded using EndNote X9 software. After the titles and abstracts were screened, 234 irrelevant publications were eliminated and 28 full-text papers were evaluated to determine which study fulfilled the qualifying requirements. Due to inconsistencies with the inclusion criteria, an additional 19 studies were removed after careful reading of the whole text. Ultimately, for this research, 9 trials [8, 15–18, 23–26] were included.  Figure 1 shows a flow diagram that summarizes the selected results.

### 3.2. Characteristics of Included Trials

Table 1 presents the baseline data from the nine studies included in our research. The publications selected were published from 2017 to 2022, with seven completed in the last 3 years. There were 359 patients in the APS cohorts and 360 patients in the control cohorts (non-APS and usual care). The type of surgery included breast conserving surgery [15], elective video-assisted thoracoscopic surgery [8], thyroid surgery [16, 24], lung cancer surgery [17], living kidney transplantation [23], spinal surgery [25], radical surgery for esophageal carcinoma [26], and cesarean section [18].

Five types of APS were used: electrical acupuncture [15], transcutaneous electrical acupoint stimulation [8, 23, 25, 26], moxibustion therapy [16], auricular point pressing [17], and acupoint massage [18, 24]. Auricular points or acupuncture points on the body were stimulated. Body points that were often employed including Shenmen (HT7), Hegu (LI4), Baihui (DU 20), Sanyinjiao (SP6), Zusanli (ST36), Sishencong (EX-HN1), and Neiguan (P6); auricular points were frequently employed including Neifenmi (CO18), Jiaogan (AH6a), Xin (CO15), Pizhixia (AT4), and Chuiqian (LO4).

Of all the included studies, three assessed the outcome with a subjective scale PSQI only [16, 17, 24], one used two types of subjective scale to evaluate sleep quality, including AIS and PSQI [26], and one used the subjective scale ISI [23]. Three studies reported the efficacy of APS on SE [8, 15, 25], and three reported the effect on TST [15, 18, 25].

### 3.3. Risk of Bias

**Figure 2:**
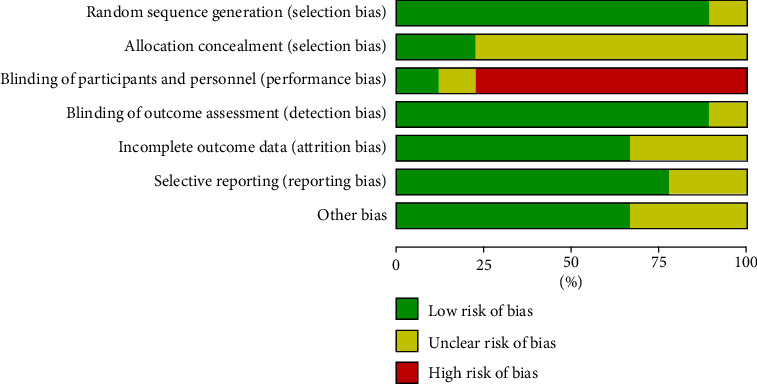
Risk of bias graph.

**Figure 3:**
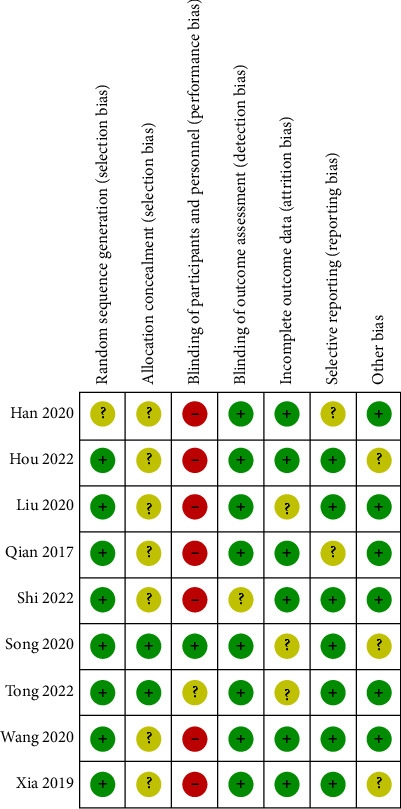
Risk of bias summary.

Figures 2 and 3 illustrate the risk of bias for every qualified research, as well as the overall risk of bias for all chosen trials. The random number table approach was employed in eight studies, which were regarded as a low selection risk [8, 15–17, 23–26]. The randomization strategy of the final one study [18] was not disclosed in detail; hence, it was considered to have an unknown selection risk. Two studies [8, 15] assessed the low selection risk of selection bias; the rest seven studies [16–18, 23–26] showed unclear risk for the lack of information on the selection bias. Most of the qualified studies had a high risk of bias among the performance bias because of the specificity of APS therapy. For detection bias, eight studies [8, 15–18, 23, 24, 26] showed low risk, and the remaining one [25] apprised unclear risk due to lack of relevant details.

### 3.4. Outcome

#### 3.4.1. Meta-Analysis Findings

**Figure 4:**
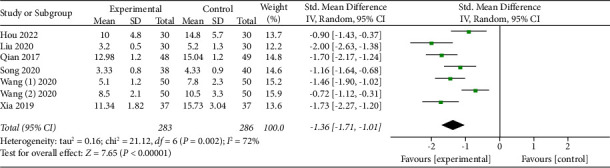
Forest plot assessing the effect of APS on perioperative subjective sleep quality.

**Figure 5:**
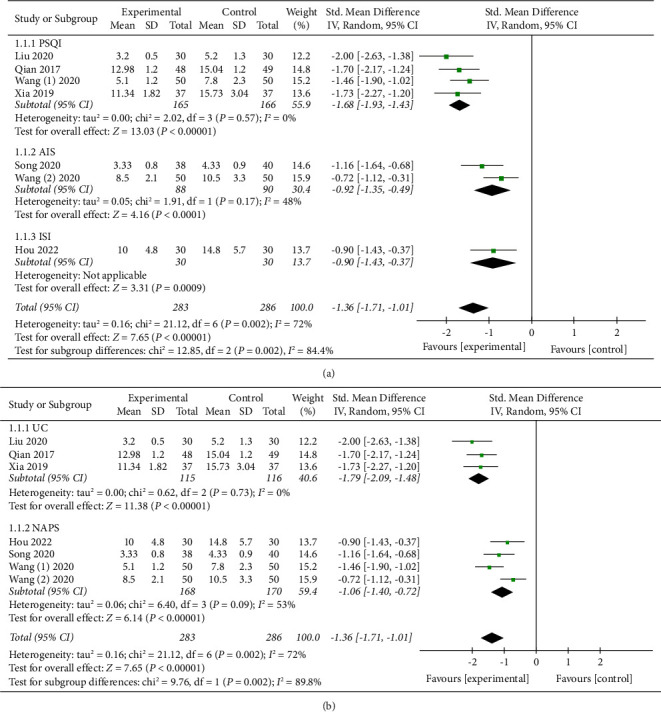
Forest plot of subgroup analysis.

**Figure 6:**
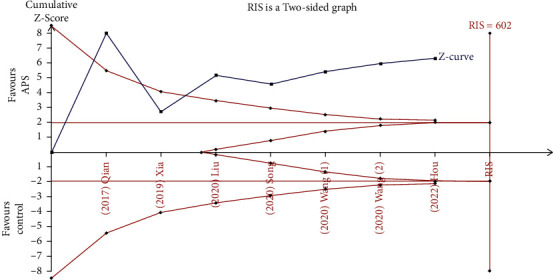
Trial sequence analysis for the effect of APS on perioperative subjective sleep quality.

The combined results of the qualified RCTs examining the efficiency of APS on sleep disorders among perioperative inpatients were presented in our meta-analysis. The main outcome of our study was subjective sleep quality. Six studies [8, 16, 17, 23, 24, 26] subjectively assessed the efficiency of APS on perioperative sleep quality. It was clear that APS could effectively enhance perioperative subjective sleep quality through synthesizing the data from the six investigations (SMD: −1.36; 95% CI: −1.71 to −1.01; *P* < 0.00001), see Figure 4. There was obvious heterogeneity among the six trials (*P* = 0.002; *I*^2^ = 72%), so we employed a random-effects model to conduct the statistical analysis. Considering that the different types of subjective scales (PSQI, AIS, and ISI) were used to evaluate the perioperative subjective sleep quality in our study, we applied subgroup analysis to detect the source of heterogeneity. The results showed that the scores of the two subjective scales, PSQI (SMD = −1.68, 95% CI: −1.93 to −1.43; *P* = 0.57, *I*^2^ = 0%) and AIS (SMD = −0.92, 95% CI: −1.35 to −0.49; *P* = 0.17, *I*^2^ = 48%), were significantly lower in the APS group, see Figure 5(a). Furthermore, a subgroup analysis was conducted based on the different Control groups, which included usual care and non-APS groups. When we excluded the results of the non-APS groups, no statistical heterogeneity was found in our data analysis (SMD = −1.79, 95% CI: −2.09 to −1.48; *P* = 0.73, *I*^2^ = 0%; 231 participants, Figure 5(b)). The credibility of the synthesized data was further verified by TSA with the *Z*-curve transcending not only the conventional boundary but also the trial sequential monitoring boundary, see Figure 6.

#### 3.4.2. Effects of APS Therapy on Perioperative TST

**Figure 7:**
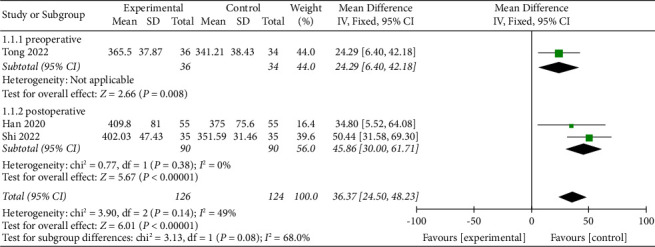
Forest plot assessing the effect of APS on perioperative TST.

Three studies [15, 18, 25] assessed the effects of APS therapy on perioperative TST, with one study [15] evaluating the TST during the preoperative period and two, [18, 25] during the postoperative period. The results suggested that, compared to the control group, TST was higher in the APS group during the preoperative period (MD = 24.29; 95% CI: 6.4 to 42.18; *P*=0.0008) and the postoperative period (MD = 45.86; 95% CI: 30.00 to 61.71; *P* < 0.00001), see Figure 7.

#### 3.4.3. Effects of APS Therapy on Perioperative SE

**Figure 8:**
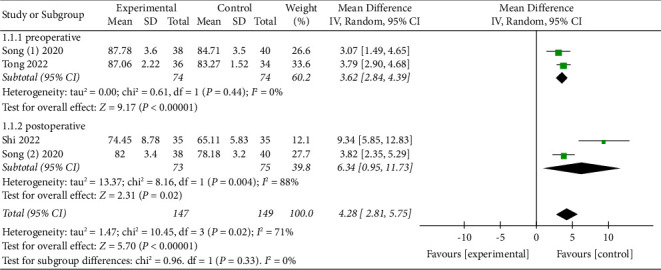
Forest plot assessing the effect of APS on perioperative SE.

Three studies [8, 15, 25] evaluated the efficacy of APS therapy on perioperative SE. One research [15] only assessed preoperative SE, one [25] only estimated postoperative SE, and the remaining one [8] evaluated both preoperative and postoperative SE. Synthesized analysis of the three studies suggested that APS therapy dramatically improved perioperative SE (preoperative MD = 3.62, 95% CI: 2.84 to 4.39, *P* < 0.00001; postoperative MD = 6.43, 95% CI: 0.95 to 11.73, *P* < 0.00001), see Figure 8.

### 3.5. Sensitivity Analysis

**Figure 9:**
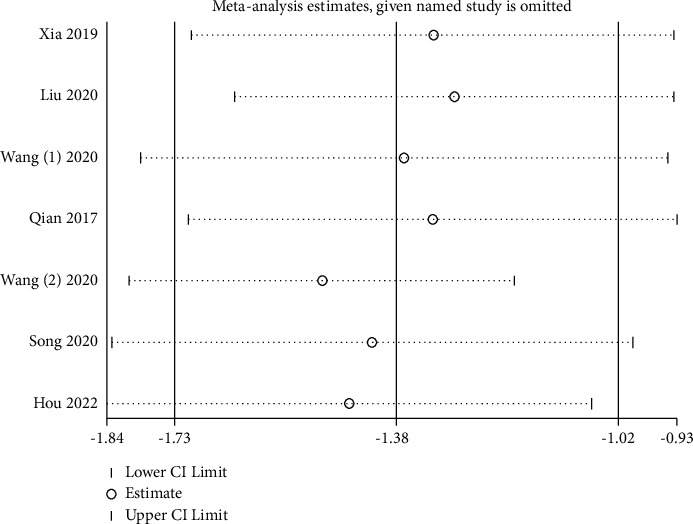
Sensitivity analysis for the effect of APS on perioperative subjective sleep quality.

A sensitivity analysis was performed to examine the impact of a single study on the total effect size. No matter which article was left out, the combined total result of the primary outcome was unaffected, proving that the main conclusion in our meta-analysis was robust, see Figure 9.

## 4. Discussion

It was frequently reported that patients experienced various kinds and severity levels of sleep problems during the perioperative period, which might last for a long time after anesthesia and surgery [27]. Patients undergoing various procedures were vulnerable to perioperative sleep disorders. Obstructive sleep apnea (OSA), decreased total sleep time, fragmented sleep, abnormal circadian rhythms, and other issues were all included in perioperative sleep disorders [3]. Several risk factors could lead to the incidence of perioperative sleep disorders, such as age [28], pain [29], mental diseases [30], and surgery [31]. Interestingly, some prior studies suggested that perioperative sleep disorders could contribute to altered pain perception and postoperative cognitive dysfunction and lead to poor recovery [3, 32]. It was suggested that patients in the intensive care unit following thoracic surgery were observed to have postoperative sleep problems, including 62% of patients within 6 months and 12% of patients at all follow-up visits experiencing poor sleep [33]. However, the prevalence of sleep disturbance was not taken seriously by medical staff for a long time. For example, almost 25% of patients undergoing cardiac surgery suffered from sleep disturbance, with 80% of these patients remaining unidentified before the operations [34]. Fortunately, with the development of perioperative medical care, perioperative sleep disorders gained more attention and various interventions emerged. The interventions could be divided into two categories: pharmacological and nonpharmacological therapy. Dexmedetomidine and melatonin were commonly used medications. Due to its sedative, antianxiety, analgesic, and anti-inflammatory effects, dexmedetomidine improved the total sleep time and sleep efficiency of hospitalized patients [35]. Melatonin, an endogenous hormone, was another major pharmacological approach in regulating the circadian rhythm. It was reported that melatonin could reduce the length of sleep latency, daytime napping, and nocturnal waking following surgery [36]. However, the traditional pharmacological therapies were often limited due to the apparent adverse reactions.

APS, a method of traditional Chinese medicine, was used as a new nonpharmacological therapy for perioperative sleep disorders recently, which was easy to learn and safe to apply [37]. A large number of RCTs examined the efficiency of APS on perioperative sleep disorders, and the results indicated that APS could significantly improve perioperative sleep quality [8]. A prior meta-analysis also concluded that APS at various acupoints might significantly increase the body's neurotransmitter levels and regulate biological clock genes to enhance patients' sleep quality during the postoperative period [37]. Despite the fact that its therapeutic efficacy was relatively definite, it was not examined quantitatively and systematically in prior studies.

We conducted the meta-analysis to analyze and confirm the results by gathering as many publications as feasible, summarizing features of the trials, and synthesizing their data. This study dealt with whether APS therapies could improve perioperative sleep disorders. We chose this topic, given that APS was frequently employed to gain better perioperative sleep quality in observational studies in recent years [8, 15]. We detected nine trials with remarkable variations in the types of APS and acupuncture points, types of surgery, study sample sizes, and research qualities. Given these biases, we carried out a comprehensive summary, accompanied by a quantitative evaluation with subgroup analysis. The pooled results (Figure 4) suggested that the APS therapies could contribute to improve perioperative sleep quality. Then, we conducted a subgroup analysis since different types of subjective scales (PSQI, AIS, and ISI) were used to estimate the efficiency of APS on perioperative sleep quality (Figure 5(a)) and identified that the differences of subjective sleep quality scales did not affect our statistical results. Nonetheless, it was still necessary to unify the assessment criteria of sleep disturbance based on the internal discrepancy among the subjective scales. For instance, the PSQI is used to evaluate the last month's sleep time, subjective sleep quality, sleep habits, sleep efficiency, sleep interruptions, usage of sleeping drugs, and daytime dysfunction [27]. As another subjective scale assessing sleep quality, the AIS is a self-rating scale that assesses sleep induction, nighttime awakenings, ultimate awakening, overall sleep duration, and sleep quality [27].

The advantages of our meta-analysis were as follows: This was the first meta-analysis to estimate the effects of APS on sleep quality in all surgical patients during the whole perioperative period. Prior studies had certain restrictions. Luo's study merely examined the efficiency of electroacupuncture on sleep quality during the postoperative period and did not conduct a quantitative meta-analysis [12]. In addition, no meta-analysis was conducted to evaluate the effects of APS on perioperative objective sleep-related indicators. Therefore, our meta-analysis combined and evaluated the qualified studies that provided objective sleep-related indicators (TST and SE), which might more accurately and objectively estimate the efficiency of APS on perioperative sleep disorders. Meanwhile, we identified two possible causes of heterogeneity, which were differences in the type of subjective scales employed and in their arrangements of the control groups. More importantly, we also performed trial sequential analysis to validate the results of our study, which made our results more valuable than the prior studies.

Our meta-analysis included some limitations that should also be taken into account. First, only articles published in Chinese and English were considered for this meta-analysis, while the studies in other languages were excluded. Second, several qualified RCTs did not precisely notify allocation concealment and reporting bias, which might cause a mild deviation in the meta-analysis results. Third, small sample sizes and the lack of follow-up evaluation in the majority of the randomized trials might cause the advantages of APS on perioperative sleep disorders to be overstated. Only six studies were included in the analysis of the main outcome, the subjective sleep quality, so further studies with a large sample size and high quality are still required.

## 5. Conclusion

Taken together, our systematic review and meta-analysis suggested that APS could effectively improve perioperative sleep disorders, indicating that it was worthwhile to offer such therapies in perioperative healthcare institutions. Although our results had certain clinical implications of APS for the treatment of perioperative sleep disorders, more studies are needed to offer stronger evidence to further verify our conclusions in the future.

## Figures and Tables

**Table 1:** Characteristics of the included studies.

Included studies	Study design	*n*(intervention/control group)	Surgery type	APS type	Control	Intervention (acupoints)	Outcomes
Tong et al. [15]	RCT	36/34	Breast-conserving surgery	EA	UC	Shenmen (HT7), Neiguan (PC6), Yintang (EX-HN3), and Baihui (DU20)	TST and SE

Song et al. [8]	RCT	38/40	Elective video-assisted thoracoscopic surgery	TEAS	NAPS	Shenmen (HT7), Neiguan (PC6), Zusanli (ST36), and Hegu (LI4)	AIS and SE

Xia et al. [16]	RCT	37/37	Thyroid cancer surgery	MT	UC	Baihui (DU 20)	PSQI

Liu [23]	RCT	30/30	Lung cancer surgery	APP	UC	Neifenmi (CO18), Jiaogan (AH6a), Xin (CO15), Pizhixia (AT4), Chuiqian (LO4), and Shenmen (TF4)	PSQI

Hou et al. [18]	RCT	30/30	Living kidney transplantation	TEAS	NAPS	Shenmen (HT7), Neiguan (PC6), and Sanyinjiao (SP6)	ISI

Shi et al. [24]	RCT	35/35	Spinal surgery	TEAS	NAPS	Shenmen (HT7), Sanyinjiao (SP6), and Baihui (DU 20)	TST and SE

Wang et al. [3]	RCT	50/50	Radical surgery for esophageal carcinoma	TEAS	NAPS	Neiguan (PC6)	AIS and PSQI
						Hegu (LI4)	

Xia [26]	RCT	55/55	Cesarean section	AM	UC	Sishencong (EX-HN1), Anmian, Sanyinjiao (SP6), Shenmen (HT7), and Shimian	TST

Qian et al. [17]	RCT	48/49	Thyroid surgery	AM	UC	Shenmen (HT7), Baihui (DU20), Shenting (DU24), Fengchi (GB20), Zusanli (ST36), Sanyinjiao (SP6), Taichong (LR3), and Yanglingquan (SP9)	PSQI

APS: acupuncture-point stimulation; UC: usual care; NAPS: nonacupuncture-point stimulation; EA: electrical acupuncture; TEAS: transcutaneous electrical acupoint stimulation; MT: moxibustion therapy; APP: auricular point pressing; AM: acupoint massage; TST: total sleep time; SE: sleep efficiency; PSQI: Pittsburgh Sleep Quality Index; AIS: Athens Insomnia Scale; ISI: Insomnia Severity Index.

## Data Availability

The data that support the findings of this study are available upon reasonable request from the corresponding author.

## Supplementary Materials

Supplementary Materials
*Supplementary 1.* Supplementary Material Search Strategy.
