# Supplementary material for: Effects of Acupuncture-Point Stimulation on Perioperative Sleep Disorders: A Systematic Review with Meta-Analysis and Trial Sequential Analysis
Source: Int J Clin Pract. 2024 Jan 4;2024:6763996. doi: 10.1155/2024/6763996 (PMC10783988; doi:10.1155/2024/6763996)
Supplement: Supplementary Materials — Supplementary 1. Supplementary Material Search Strategy. [file 6763996.f1.docx]

**Supplementary 1**

**Supplementary Material Search Strategy**

**Chinese databases：**

| **Chinese databases** | Retrieval type |
| --- | --- |
| CNKI | ( ( ( Subject %='Sleep' or Title%='Sleep' ) AND ( Subject%='Sleep' or Title%='Sleep' ) AND ( Subject %='Surgery' or Title%='Surgery' ) ) ) ) ) AND ( ( Subject %='Sleep' or Title%='Sleep' ) AND ( Subject %='Surgery' or Title%='Surgery' ) ) AND ( Subject %='Acupuncture Points' or Title%='Acupuncture Points' ) ) ) AND ( Subject%='Sleep' or Title%='Sleep' ) AND ( Subject%='Surgery' or Title%='Surgery' ) ) AND ( Subject%='Acupuncture Points' or Title%='Acupuncture Points' ) ) AND ( Abstract='Random' ) ) |
| WanFang | ((Subject=Sleep) AND Subject=Surgery) AND Subject=Acupuncture Points) AND Topic = Random |
| VIP | (((Title or keyword = perioperative OR title or keyword = perioperative) OR title or keyword = perioperative period) OR title or keyword = perioperative period) OR title or keyword = preoperative) OR title or keyword = postoperative) AND title or keyword = sleep) AND ((title or keyword = acupoint OR title or keyword = acu point) OR Title or keyword = acupoint) OR title or keyword = acupoints) OR title or keyword = acupuncture point) OR title or keyword = meridian point) OR title or keyword = point) OR title or keyword = points) OR title or keyword = acupuncture points) OR title or keyword = acupuncture point) OR title or keyword = acupuncture point)) AND abstract = random) |
| CBM | "sleep" [weighted: expanded] AND "perioperative" [common field: intelligence] AND "acupuncture points" [common field: intelligence] AND "random" [common field: intelligence] |

**English databases**

**Pubmed:**

Part 1 – Sleep Terms

(("Sleep"[Mesh]) OR (((((sleep*[Title/Abstract]) OR (Sleeping Habit*[Title/Abstract])) OR (insomni*[Title/Abstract])) OR (night*[Title/Abstract])) OR (circadian[Title/Abstract])))) NOT ((("Sleep Apnea Syndromes"[Mesh]) OR ((((((((((((((Sleep Apnea Syndrome*[Title/Abstract]) OR (Apnea Syndrome*[Title/Abstract])) OR (Sleep Hypopnea*[Title/Abstract])) OR (Hypopnea*, Sleep[Title/Abstract])) OR (Apnea*, Sleep[Title/Abstract])) OR (Sleep Apnea*[Title/Abstract])) OR (Sleep Apnea, Mixed Central and Obstructive[Title/Abstract])) OR (Mixed Central and Obstructive Sleep Apnea[Title/Abstract])) OR (Sleep Apnea*, Mixed[Title/Abstract])) OR (Mixed Sleep Apnea*[Title/Abstract])) OR (Hypersomnia with Periodic Respiration[Title/Abstract])) OR (Sleep-Disordered Breathing[Title/Abstract])) OR (Breathing, Sleep-Disordered[Title/Abstract])) OR (Sleep Disordered Breathing[Title/Abstract]))))

Part 2 – Acupuncture Points Terms

((((((((Acupuncture Points [MeSH Terms]) OR (Acupuncture Point[Title/Abstract])) OR (Point, Acupuncture[Title/Abstract])) OR (Points, Acupuncture[Title/Abstract])) OR (Acupoints[Title/Abstract])) OR (Acupuncture Therapy[Title/Abstract])) OR (transcutaneous electrical acupoint stimulation[Title/Abstract])) OR (TEAS[Title/Abstract])) OR (Acupuncture[Title/Abstract])

Part 3 – Surgery Terms

(("Perioperative Period"[Mesh]) OR ((((((Period*, Perioperative [Title/Abstract]) OR (Postoperative Period [Title/Abstract])) OR (surgery [Title/Abstract])) OR (Intraoperative Period [Title/Abstract])) OR (Preoperative Period [Title/Abstract])) OR (anesthesia[Title/Abstract]))))

Part 4 – Sleep Interventions and Acupuncture Points Terms combined

Part 1 AND Part 2

Part 5 – Sleep Interventions, 1Acupuncture Points and Surgery Terms combined

Part 4 AND Part 3

Part 6 – Random Controlled Trials limit applied to Part 5

**Embase**

**Query**

**Results**

**59**

**#31**

**#8** AND **#15** AND **#24** AND **#30**

**1,235,340**

**#30**

**#25** OR **#26** OR **#27** OR **#28** OR **#29**

**53,387**

**#29**

**'rct'**:ti,ab,kw

**918,345**

**#28**

**'randomized'**:ti,ab,kw

**24,128**

**#27**

**'controlled clinical trial'**:ti,ab,kw

**137,227**

**#26**

**'randomized controlled trial'**:ti,ab,kw

**745,150**

**#25**

**'randomized controlled trial'**/exp

**62,730**

**#24**

**#16** OR **#17** OR **#18** OR **#19** OR **#20** OR **#21** OR **#22** OR **#23**

**3,490**

**#23**

**'teas'**:ti,ab,kw

**250**

**#22**

**'transcutaneous electrical acupoint stimulation'**:ti,ab,kw

**2,288**

**#21**

**'acupuncture therapy'**:ti,ab,kw

**8,314**

**#20**

**'acupoint*'**:ti,ab,kw

**1,023**

**#19**

**'acupuncture point'**:ti,ab,kw

**4,717**

**#18**

**'acupuncture point'**/exp

**38,012**

**#17**

**'acupuncture'**:ti,ab,kw

**55,305**

**#16**

**'acupuncture'**/exp

**2,969,253**

**#15**

**#9** OR **#10** OR **#11** OR **#12** OR **#13** OR **#14**

**247,346**

**#14**

**'anesthesia'**:ti,ab,kw

**1,981,349**

**#13**

**'surgery'**:ti,ab,kw

**51,617**

**#12**

**'postoperative period'**:ti,ab,kw

**463,345**

**#11**

**'preoperative'**:ti,ab,kw

**184,000**

**#10**

**'perioperative'**:ti,ab,kw

**1,116,609**

**#9**

**'perioperative period'**/exp

**491,755**

**#8**

**#1** OR **#2** OR **#3** OR **#4** OR **#5** OR **#6** OR **#7**

**14,526**

**#7**

**'sleep deprivation'**:ti,ab,kw

**17,912**

**#6**

**'sleep disturbance'**:ti,ab,kw

**8,668**

**#5**

**'sleep disorder'**:ti,ab,kw

**67**

**#4**

**'sleep qualities'**:ti,ab,kw

**34,009**

**#3**

**'sleep quality'**/exp

**312,736**

**#2**

**'sleep'**:ti,ab,kw

**491,755**

**#1**

**'sleep'**/exp OR **'sleep'**

**Cochrane:**


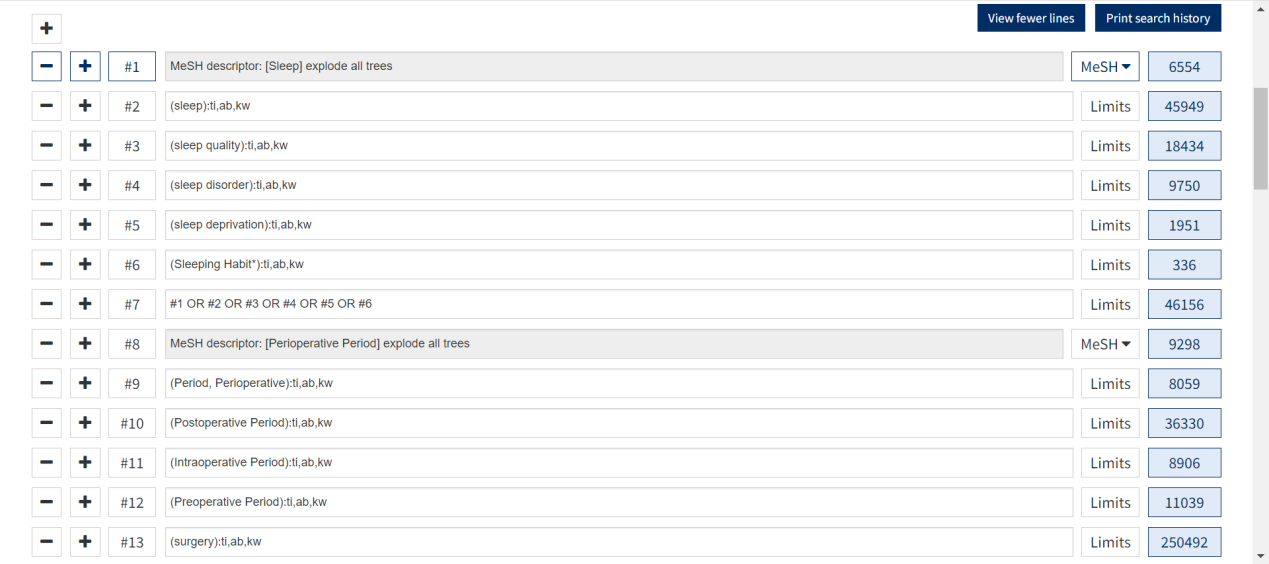


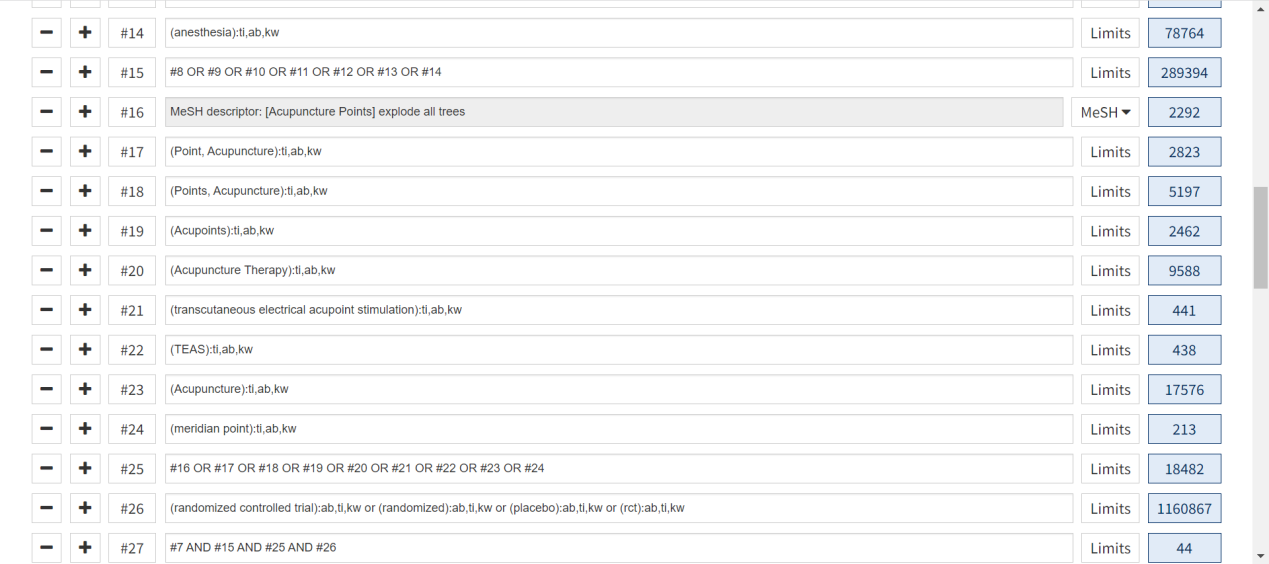


**Web of science：**

1: TS=(Sleep or Sleep Quality or Sleep Wake Disorder or sleep* or sleep deprivation or Sleeping Habit* )

2: TS=(Perioperative Period or Period*, Perioperative or Postoperative Period or Preoperative Period or anaesthesia or surgery )

3: TS=(Acupuncture Points or Point, Acupuncture or Points, Acupuncture or Acupoints or Acupuncture Therapy or transcutaneous electrical acupoint stimulation or TEAS or Acupuncture or meridian point )

4: TS=(randomized controlled trial or randomized or placebo or RCT)

5: #1 AND #2 AND #3 AND #4
